# Inhibition of Dipeptidyl Peptidase-4 by Flavonoids: Structure–Activity Relationship, Kinetics and Interaction Mechanism

**DOI:** 10.3389/fnut.2022.892426

**Published:** 2022-05-12

**Authors:** Junkun Pan, Qiang Zhang, Chunling Zhang, Wenbo Yang, Hui Liu, Zhenzhen Lv, Jiechao Liu, Zhonggao Jiao

**Affiliations:** Zhengzhou Fruit Research Institute, Chinese Academy of Agricultural Sciences, Zhengzhou, China

**Keywords:** flavonoid, dipeptidyl peptidase-4, inhibition, structure-activity relationship, kinetics, binding, interaction, molecular docking

## Abstract

With the aim to establish a structure-inhibitory activity relationship of flavonoids against dipeptidyl peptidase-4 (DPP-4) and elucidate the interaction mechanisms between them, a pannel of 70 structurally diverse flavonoids was used to evaluate their inhibitory activities against DPP-4, among which myricetin, hyperoside, narcissoside, cyanidin 3-O-glucoside, and isoliquiritigenin showed higher inhibitory activities in a concentration-dependent manner. Structure-activity relationship analysis revealed that introducing hydroxyl groups to C3', C4', and C6 of the flavonoid structure was beneficial to improving the inhibitory efficacy against DPP-4, whereas the hydroxylation at position 3 of ring C in the flavonoid structure was unfavorable for the inhibition. Besides, the methylation of the hydroxyl groups at C3', C4', and C7 of the flavonoid structure tended to lower the inhibitory activity against DPP-4, and the 2,3-double bond and 4-carbonyl group on ring C of the flavonoid structure was essential for the inhibition. Glycosylation affected the inhibitory activity diversely, depending on the structure of flavonoid aglycone, type of glycoside, as well as the position of substitution. Inhibition kinetic analysis suggested that myricetin reversibly inhibited DPP-4 in a non-competitive mode, whereas hyperoside, narcissoside, cyanidin 3-O-glucoside, and isoliquiritigenin all reversibly inhibited DPP-4 in a mixed type. Moreover, the fluorescence quenching analysis indicated that all the five flavonoid compounds could effectively quench the intrinsic fluorescence of DPP-4 by spontaneously binding with it to form an unstable complex. Hydrogen bonds and van der Waals were the predominant forces to maintain the complex of myricetin with DPP-4, and electrostatic forces might play an important role in stabilizing the complexes of the remaining four flavonoids with DPP-4. The binding of the tested flavonoids to DPP-4 could also induce the conformation change of DPP-4 and thus led to inhibition on the enzyme. Molecular docking simulation further ascertained the binding interactions between DPP-4 and the selected five flavonoids, among which hyperoside, narcissoside, cyaniding 3-O-glucoside, and isoliquiritigenin inserted into the active site cavity of DPP-4 and interacted with the key amino acid residues of the active site, whereas the binding site of myricetin was located in a minor cavity close to the active pockets of DPP-4.

## Introduction

Diabetes mellitus (DM) is a serious global health issue with increasing prevalence in recent years. According to the International Diabetes Federation, there were 463 million of adults aged 20–79 years living with diagosed or undiagosed diabetes in 2019, and this number was expected to rise to 578 million by 2030 and 700 million by 2045 (1). Type 2 diabetes mellitus (T2DM) is the most prevalent form of diabetes, accounting for over 90% of all cases (2). It is characterized by impaired insulin secretion and increased insulin resistance, which lead to elevated blood glucose levels (3, 4). Chronic hyperglycemia can further induce kidney disease, atherosclerosis, heart dysfunction and other harmful complications (5, 6), making a serious impact on the lives and wellbeing of individuals as well as their families.

The common strategies for the treatment of T2DM include delaying gastric emptying and absorption of carbohydrates, enhancing insulin signaling and increasing insulin secretion. Glucagon-like peptide-1 (GLP-1) and glucose-dependent insulinotropic peptide (GIP) are gut-derived incertin hormones that can stimulate insulin secretion, and thereby lower postprandial blood glucose levels (7, 8). GLP-1 can also lower plasma glucose levels through prolonging gastric emptying, reducing glucagon secretion and suppressing food intake (7). However, the incertins in the circulation can be inactivated rapidly by dipeptidyl peptidase-4 (DPP-4, EC 3.4.14.5), a serine protease widely expressed in numerous tissues, thus that the actions are terminated (9–11). Inhibition of DPP-4 can prevent the degradation of incertins and thereby enhancing their actions, providing an attractive alternative approach for the treatment and prevention of T2DM (12). To date, more than ten inhibitors of DPP-4, such as sitagliptin, vildagliptin, saxagliptin, linagliptin, and alogliptin, have been approved as therapeutic options for the management of patients with T2DM (12, 13). However, these synthetic DPP-4 inhibitors have been shown to be associated with some adverse effects such as increased risk of heart failure (14), allergic reactions (15), polyarthritis (16), bullous pemphigoid (17). Thus, it is necessary to develop potential DPP-4 inhibitors with higher safety and fewer adverse effects from natural resources.

Flavonoids exist abundantly in various plants and exhibit a wide range of bioactivities and potential benefits to human health (18, 19). Many literatures have documented the anti-diabetic effects of various flavonoid compounds (20, 21). The flavonoid compounds can prevent the digestion of carbohydrates and production of glucose by inhibiting the activities of α-amylase and α-glucosidase (22), retard glucose absorption by inhibiting glucose transporters (23), ameliorate insulin resistance by modulating insulin signaling pathway and inhibiting inflammatory signaling (24, 25). Notably, it was found that some flavonoid compounds have the capacity to modulate the activity of DPP-4 (26), which gives a new insight into the mechanism involved in the anti-diabetic activity of flavonoids. Nevertheless, the results obtained from different experiments are diverse and variable probably due to the variability of test methods and conditions (26, 27), which makes it difficult to compare and consequently achieve conclusions about the inhibitory efficacy against DPP-4. A large panel of flavonoids for evaluation of the inhibitory activities against DPP-4 may be helpful for establishing an accurate and adequate structure-activity relationship (SAR) and screening for active flavonoids for investigation of the inhibition kinetics, binding mode as well as the molecular mechanisms. Besides, few researches can be found regarding the inhibition kinetics and interaction mechanisms of flavonoids against DPP-4.

With the aim to establish a structure-inhibitory activity relationship of flavonoids against DPP-4 and elucidate the interaction mechanisms between them, a pannel of 70 structurally diverse flavonoids was collected and divided into seven groups according to their structural features, and then their abilities for inhibiting the activity of DPP-4 were evaluated by using a fluorescence method. The selected flavonoids with higher inhibitory activity against DPP-4 were used for further researches to characterize the inhibition kinetics and interaction mechanisms.

## Materials and Methods

### Chemicals

DPP-4 (human recombinant, No. D3446) and Gly-Pro-7-amido-4-methylcoumarin hydrobromide (Gly-Pro-AMC, CAS 115035-46-6) were purchased from Sigma Aldrich (St. Louis, MO, U.S.A.). Flavonoid standards were obtained from Shanghai Yuanye Bio-Technology Co., Ltd. (Shanghai, China). Sitagliptin phosphate monohydrate, dimeththyl sulfoxide (DMSO), and Tris-HCl buffer solution (1.0 M, pH 8.0) were supplied by Beijing Solarbio Science & Technology Co., Ltd. (Beijing, China).

### *In vitro* Assay for DPP-4 Inhibitory Activity

The DPP-4 inhibitory activities of flavonoids were determined according to the methods described in previous reports (28, 29) with slight modifications. Briefly, 26 μL of the tested flavonoids dissolved in DMSO and 24 μL of DPP-4 (1.73 mU/mL) dissolved in Tris-HCl buffer solution (50 mM, pH 8.0) were mixed in a 96-well microplate and incubated at 37 °C for 10 min. Then, 50 μL of the DPP-4 substrate (Gly-Pro-AMC, 200 mM) dissolved in Tris-HCl buffer solution (50 mM, pH 8.0) was added into each reaction well and the resulting mixture was incubated at 37 °C for 30 min. The enzymatic reaction was monitored during the incubation by measuring the fluorescence (*FLU*, λ_*ex*_=360/λ_*em*_=460 nm) in kinetic mode with a microplate reader (SpectraMax i3x, Molecular Devices, U.S.A.). The inhibitory activity was calculated using the slope of Δ*FLU*/min between 15 and 30 min, and the results were reported as % inhibition of DPP-4 activity.

### Inhibition Kinetic Analysis

Selected flavonoids with higher inhibitory activity were used to perform inhibition kinetic analysis. Firstly, the inhibitory effects of the selected flavonoids at different concentrations of DPP-4 (0.21–0.84 mU/mL) and 200 μM of Gly-Pro-AMC were measured, and the enzymatic reaction rate (*V*) vs. concentration of DPP-4 at different concentrations of inhibitor was plotted to demonstrate the reversibility of the inhibition. For a reversible inhibition, the inhibition type (competitive, uncompetitive, non-competitive or mixed) of the tested flavonoid compound was further determined by using Lineweaver-Burk plot as well as Dixon and Cornish-Bowden plots according to the methods described in previous researches (30–32). The concentration range of the substrate (Gly-Pro-AMC) was 50–200 μM, and the final concentration of DPP-4 was 0.42 mU/mL. The maximum enzymatic reaction rate (*V*_*max*_) and Michaelis constant (*K*_*m*_) were calculated according to the Lineweaver-Burk curve, and the competitive inhibition constant (*K*_*ic*_) and uncompetitive inhibition constant (*K*_*iu*_) were calculated according to the Dixon equation and Cornish-Bowden equation, respectively.

### Fluorescence Quenching Experiments

The fluorescence spectra of DPP-4 in the absence or presence of flavonoids were measured at 298K and 310K, respectively, by using a F7000 spectrofluorometer (HITACHI, Japan) at an excitation wavelength of 280 nm. The emission spectra were recorded in a scanning wavelength range of 290–500 nm. The excitation and emission bandwidths were both set to 2.5 nm. The final concentration of DPP-4 was 4.22 mU/mL for each measurement, and all the mixtures were allowed to equilibrate for 5 min before measurement. The synchronous fluorescence spectra were measured at 298 K by setting the excitation and emission wavelength interval (Δλ) to 15 and 60 nm, respectively. All the fluorescence spectra were corrected by subtracting the background intensities of the corresponding flavonoid solution. Since the ultraviolet absorption of the tested flavonoids at the excitation and emission wavelengths might interfere with the fluorescence intensity of DPP-4, the fluorescence data were finally corrected by using the following equation to eliminate the “inner filtering effect” (33):


$$\begin{array}{l} F_{c}={F_{m}e}^{\left(\text{Aex}+\text{Aem}\right)/2} \end{array}$$

Where *F*_*c*_ and *F*_*m*_ are the fluorescence intensities corrected and measured, respectively, while *Aex* and *Aem* are the absorbance of the tested flavonoids at the excitation and emission wavelengths, respectively.

### Molecular Docking

The crystal structure of DPP-4 (PDB ID: 1X70) was downloaded from the Research Collaboratory for Structural Bioinformatics Protein Data Bank (RCSB PDB). Before docking simulation, the structure of DPP-4 was prepared by removing the water, ligands and other irrelevant small molecules, adding hydrogen atoms and charge, repairing residues. The mol2 formats of the tested flavonoids were downloaded from PubChem database and the structures were prepared by minimizing the energy, removing the water, adding atomic charge, assigning atomic type, and making all flexible bonds rotatable. Molecular docking simulation was performed by using AutoDock 4.2 software, and the docking model of a complex with the lowest binding energy for each flavonoid was considered to be the most favorable binding mode. The interactions between the flavonoids and DPP-4 were visualized with Pymol software.

### Statistical Analysis

Statistical analysis was performed by using Origin 8.0 software and the results were presented as mean ± standard deviation (SD) for three independent experiments. *P* < 0.05 was considered to be statistically significant.

## Results and Discussion

### *In vitro* Inhibitory Effects of Flavonoids Against DPP-4

A panel of 70 flavonoids with different structures was evaluated for their *in vitro* inhibitory activities against DPP-4 by using the fluorescence method. As shown in Table 1, only five flavonoids, namely hyperoside (E4), myricetin (E19), narcissoside (E21), cyanidin 3-O-glucoside (F2), isoliquiritigenin (G1), exhibited an inhibitory percentage of higher than 50% at the test concentration of 200 μM. The IC_50_ values of these flavonoids were determined to be 138.79 ± 0.87 μM, 156.29 ± 1.18 μM, 166.52 ± 0.6 μM, 81.05 ± 4.14 μM, and 149.96 ± 0.58 μM, respectively. Gao et al. (34) also reported a strong inhibitory activity of cyanidin 3-O-glucoside against DPP-4 with a IC_50_ value of 8.26 μM in their experiments. The difference in the IC_50_ value might be owing to the use of different DPP-IV inhibitor screening assay kit, in which the origin of DPP-4, concentration of the enzyme and substrate may be different from each other, and thus leading to differences in the obtained results among different researches. In an experiment for evaluation of the *in vitro* DPP-4 inhibitory activity of 45 structurally related flavonoids by Proença et al. (29), only three flavonoids showed an inhibitory percentage of higher than 50% at the test concentration of 200 μM, and the IC_50_ value of the most active flavonoid were 73 ± 2 μM by using a fluorometric method. The inhibitory effects of several flavonoids, such as apigenin, luteolin, chrysin, baicalein, kampferol 4'-O-glucoside, rutin, and galangin, obtained from the above research, were all similar to our research. Notably, hyperoside and isoliquiritigenin were identified as potential strong DPP-4 inhibitors for the first time in the present research as compared to other flavonoids.

**Table 1:** Inhibitory effects of flavonoids against DPP-4.

**No**.	**Flavonoids**	**Skeleton**	**C3**	**C5**	**C6**	**C7**	**C8**	**C2'**	**C3'**	**C4'**	**C5'**	**% Inhibition at 200μM**
A1	Apigenin	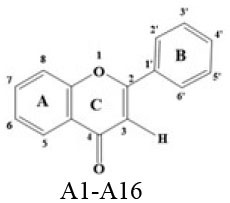	H	OH	H	OH	H	H	H	OH	H	46.36 ± 1.49
A2	Baicelein		H	OH	OH	OH	H	H	H	H	H	39.38 ± 0.41
A3	Chrysin		H	OH	H	OH	H	H	H	H	H	26.08 ± 3.07
A4	Diosmetin		H	OH	H	OH	H	H	OH	OMe	H	22.20 ± 1.22
A5	Diosmin		H	OH	H	ORut	H	H	OH	OMe	H	16.35 ± 1.73
A6	Isoorientin		H	OH	Glu	OH	H	H	OH	OH	H	42.63 ± 0.33
A7	Isovitexin		H	OH	Glu	OH	H	H	H	OH	H	42.36 ± 1.23
A8	Luteolin		H	OH	H	OH	H	H	OH	OH	H	47.51 ± 0.89
A9	Luteolin 7-O-glucuronide		H	OH	H	OGlc	H	H	OH	OH	H	35.65 ± 0.11
A10	Luteoloside		H	OH	H	OGlu	H	H	OH	OH	H	45.61 ± 0.62
A11	Narirutin		H	OH	H	ORut	H	H	H	OH	H	13.97 ± 0.13
A12	Orientin		H	OH	H	OH	Glu	H	OH	OH	H	38.61 ± 2.10
A13	Tangeretin		H	OMe	OMe	OMe	OMe	H	H	OMe	H	14.16 ± 1.06
A14	Vitexin		H	OH	H	OH	Glu	H	H	OH	H	27.01 ± 1.35
A15	Vitexin 2”-glucoside		H	OH	H	OH	Glg	H	H	OH	H	36.97 ± 0.63
A16	Vitexin 4'-O-glucoside		H	OH	H	OH	Glu	H	H	OGlu	H	45.90 ± 1.48
B1	Biochanin A	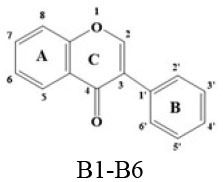	H	OH	H	OH	H	H	H	OMe	H	14.35 ± 1.48
B2	Calycosin		H	H	H	OH	H	H	OH	OMe	H	42.99 ± 2.41
B3	Daidzein		H	H	H	OH	H	H	H	OH	H	19.25 ± 3.44
B4	Formononetin		H	H	H	OH	H	H	H	OMe	H	17.56 ± 0.19
B5	Genistein		H	OH	H	OH	H	H	H	OH	H	15.31 ± 0.21
B6	Genkwanin		H	OH	H	OMe	H	H	H	OH	H	20.78 ± 1.92
C1	Dihydromorin	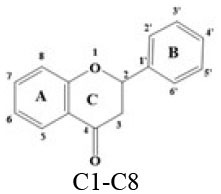	OH	OH	H	OH	H	OH	H	OH	H	30.92 ± 1.22
C2	Dihydromyricetin		OH	OH	H	OH	H	H	OH	OH	OH	20.59 ± 0.36
C3	Hesperetin		H	OH	H	OH	H	H	OH	OMe	H	<10
C4	Hesperidin		H	OH	H	ORut	H	H	OH	OMe	H	<10
C5	Liquiritigenin		H	H	H	OH	H	H	H	OH	H	17.06 ± 0.94
C6	Naringenin		H	OH	H	OH	H	H	H	OH	H	<10
C7	Neohesperidin		H	OH	H	ONeo	H	H	OH	H	OMe	11.79 ± 1.61
C8	Taxifolin		OH	OH	H	OH	H	H	OH	OH	H	19.93 ± 0.65
D1	Catechin	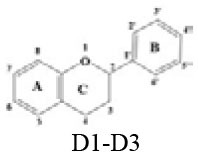	OH	OH	H	OH	H	H	OH	OH	H	<10
D2	Epicatechin		OH	OH	H	OH	H	H	OH	OH	H	<10
D3	Epigallocatechin gallate		Gallate	OH	H	OH	H	H	OH	OH	OH	<10
E1	Avicularin		OAra	OH	H	OH	H	H	OH	OH	H	27.10 ± 0.42
E2	Galangin	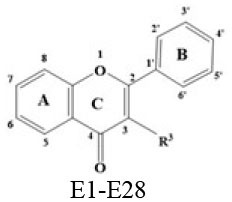	OH	OH	H	OH	H	H	H	H	H	38.26 ± 0.36
E3	Herbacetin		OH	OH	H	OH	H	H	H	OH	H	25.74 ± 1.82
**E4**	**Hyperoside**		**OGla**	**OH**	**H**	**OH**	**H**	**H**	**OH**	**OH**	**H**	**62.10** **±0.16**
E5	Isoquercitrin		OGlu	OH	H	OH	H	H	OH	OH	H	36.94 ± 1.13
E6	Isorhamnetin		OH	OH	H	OH	H	H	OMe	OH	H	29.35 ± 1.52
E7	Isorhamnetin 3-O-glucoside		Glu	OH	H	OH	H	H	OMe	OH	H	38.58 ± 1.41
E8	Isorhamnetin 3-O-neohesperidin		ONeo	OH	H	OH	H	H	OMe	OH	H	40.63 ± 0.42
E9	Kaempferide		OH	OH	H	OH	H	H	H	OMe	H	15.68 ± 0.58
E10	Kaempferitrin		Dir	OH	H	Dir	H	H	H	OH	H	19.27 ± 0.47
E11	Kaempferol		OH	OH	H	OH	H	H	H	OH	H	32.23 ± 0.19
E12	Kaempferol 3-O-arabinoside		OAra	OH	H	OH	H	H	H	OH	H	39.24 ± 0.43
E13	Kaempferol 3-O-galactoside		OGal	OH	H	OH	H	H	H	OH	H	46.32 ± 1.57
E14	Kaempferol 3-O-rutinoside		ORut	OH	H	OH	H	H	H	OH	H	36.83 ± 0.01
E15	Kaempferol 3-O-sophoroside		OSop	OH	H	OH	H	H	H	OH	H	43.19 ± 1.48
E16	Kaempferol 4'-O-glucoside		OH	OH	H	OH	H	H	H	OGlu	H	37.22 ± 0.64
E17	Kaempferol 7-O-glucoside		OH	OH	H	OGlu	H	H	H	OH	H	48.84 ± 1.07
E18	Morin		OH	OH	H	OH	H	OH	H	OH	H	42.79 ± 0.91
**E19**	**Myricetin**		**OH**	**OH**	**H**	**OH**	**H**	**H**	**OH**	**OH**	**OH**	**53.16** **±0.72**
E20	Myricitrin		ORha	OH	H	OH	H	H	OH	OH	OH	39.8 ± 0.78
**E21**	**Narcissoside**		**ORut**	**OH**	**H**	**OH**	**H**	**H**	**OMe**	**OH**	**H**	**57.50** **±1.28**
E22	3-O-Methylquercetin		OMe	OH	H	OH	H	H	OH	OH	H	45.62 ± 0.78
E23	Quercetin		OH	OH	H	OH	H	H	OH	OH	H	39.37 ± 0.58
E24	Quercetin 3-O-glu-7-O-gentiobioside		OGlu	OH	H	OGen	H	H	OH	OH	H	33.92 ± 1.07
E25	Quercetin 7-O-rhamnoside		OH	OH	H	ORha	H	H	OH	OH	H	48.96 ± 0.12
E26	Quercitrin		ORha	OH	H	OH	H	H	OH	OH	H	47.65 ± 0.36
E27	Rhamnetin		OH	OH	H	OMe	H	H	OH	OH	H	22.24 ± 0.63
E28	Rutin	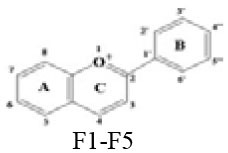	ORut	OH	H	OH	H	H	OH	OH	H	42.67 ± 1.28
F1	Cyanidin		OH	OH	H	OH	H	H	OH	OH	H	18.82 ± 0.40
**F2**	**Cyanidin 3-O-glucoside chloride**		**OGlu**	**OH**	**H**	**OH**	**H**	**H**	**OH**	**OH**	**H**	**68.05** **±0.15**
F3	Malvidin 3-O-glucoside		OGlu	OH	H	OH	H	H	OMe	OH	OMe	14.98 ± 2.42
F4	Malvin		OGlu	OGlu	H	OH	H	H	OMe	OH	OMe	13.71 ± 2.07
F5	Pelargonidin	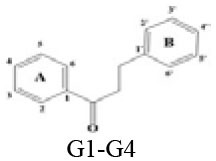	OH	OH	H	OH	H	H	H	OH	H	<10
**G1**	**Isoliquiritigenin**		**H**	**H**	**OH**	**-**	**-**	**H**	**H**	**OH**	**H**	**62.81** **±0.13**
G2	Neosperidin dihydrochalcone		H	H	OH	-	-	H	OH	OMe	H	17.15 ± 0.23
G3	Phloretin		H	H	OH	-	-	H	H	OH	H	17.80 ± 1.21
G4	Phlorizin		H	H	OH	-	-	H	H	OH	H	<10

*Me-methyl; Glu-glucoside/glucuronide; Glg-glucosylglucoside; Rut-rutinoside; Neo-neohesperidoside; Dir-dirhamnoside; Sop-sophoroside; Rha-rhamnoside; Gen-gentiobioside*.

### Stucture-Activity Relationship of Flavonoids on DPP-4 Inhibition

#### Hydroxylation and Methylation

As shown in Table 1, baicalein (A2) exhibited higher inhibitory activity than chrysin (A3), indicating that hydroxylation at position 6 of ring A might increase the inhibitory activity against DPP-4. Similar trends were found in ring B by comparing apigenin (A1) with chrysin (A3), calycosin (B2) with formononetin (B4), myricetin (E19) with quercetin (E23) and kaempferol (E11). It had been suggested that the increasing number of hydroxyl group in the flavonoid structure might be favorable for DPP-4 inhibition (27). Our research agrees partly with this. In this sense, introducing hydroxyl groups to the positions 3' and 4' of ring B as well as the position 6 of ring A seemed to be more favorable for DPP-4 inhibition, whereas the hydroxylation at position 3 of ring C showed lower inhibitory effect as evidenced by comparing the inhibitory activity of kaempferol (E11) and apigenin (A1). Moreover, luteolin (A8) and kaempferol (E11), which bear a hydroxyl group at position 4' of ring B, showed higher inhibitory activities than diosmetin (A4) and kaempferide (E9), which bear a methoxyl group at position 4' of ring B, respectively, and the inhibitory percentage of quercetin (E23, bearing a hydroxyl group at position 7 of ring A) was 1.77 times higher than that of rhamnetin (E27, bearing a methoxyl group at position 7 of ring A). The inhibitory percentage of isorhamnetin (E6, bearing a methoxyl group at position 3' of ring B) was also quite lower than that of quercetin (E23, bearing a hydroxyl group at position 3' of ring B). These results also confirmed the importance of hydroxyl groups on DPP-4 inhibition of flavonoids and indicated that methylation of the hydroxyl groups on ring A and B is not favorable for the DPP-4 inhibition of flavonoids. Gao et al. also found methylation of the hydroxyl group at position 4' of ring B led to weaker inhibitory activity against DPP-4 by comparing the inhibitory activity of eriodictyol and hesperitin (34). Nontheless, methylation of the hydroxyl group at position 3 of ring C tended to improve the inhibitory effect as evidenced by comparing the inhibitory activity of quercetin (E23) with 3-O-methylquercetin (E22). This might be ascribed to the strong electron-donating capacity of methoxyl groups, according to the speculation that position 3 of ring C favored bulky and electron-donating substituents for DPP-4 inhibition (34).

#### Glycosylation

The flavonoids in nature usually occur as glycosylated forms. The conjugation of bulky sugar groups to the core structure of flavonoids might decrease their binding affinity to DPP-4 due to steric hindrance, leading to lower inhibitory activity of flavonoid glycosides (34, 35). In this sense, the glycosides of luteolin, including luteoloside (A10), luteolin 7-O-glucuronide (A9), orientin (A12), and isoorientin (A6), all showed lower inhibitory activity than luteolin (A8), and the inhibitory percentage of apigenin (A1) were also higher than those of its glycosides, which includes vitexin (A14), vitexin 4'-O-glucoside (A16), vitexin 2”-glucoside(A15), isovitexin (A7), and narirutin (A11). Similar result was also obtained by comparing the inhibitory activity of myricetin (E19) with myricitrin (E20). However, the glycoside substituents can provide more hydroxyl groups, which might enhance the binding affinity to DPP-4, resulting in an increase in inhibitory activity for flavonoid glycosides as compared with their corresponding aglycones (27). In the present research, the inhibitory percentage of kaempferol (E11) was lower than those of its glycosides, including kaempferol 4'-O-glucoside (E16), kaempferol 7-O-glucoside (E17), kaempferol 3-O-rutinoside (E14), kaempferol 3-O-sophoroside (E15), kaempferol 3-O-arabinoside (E12), and kaempferol 3-O-galactoside (E13). The glycosylation of isorhamnetin also showed a beneficial effect for DPP-4 inhibition by comparing the inhibitory activity of isorhamnetin (E6) and its glycosides, which include isorhamnetin 3-O-glucoside (E7), isorhamnetin 3-O-neohesperidin (E8), and narcissoside (E21). The inhibitory percentage of cyanidin 3-O-glucoside (F2) was 3.62 times higher than that of cyanidin (F1). In these cases, the effects of the increased number of hydroxyl groups generated by glycoside substituents in the flavonoid structure might offset the influence of steric hindrance, leading to favorable environments for DPP-4 binding and inhibition. As for quercetin, the glycosylation at position 3 or 7 with rhamnoside (E26), galactoside (E4), rhamnoside (E25), and rutinoside (E28) increased the inhibitory activity, whereas glycosylation with glucoside (E5), arabinoside (E1), glucoside and gentiobioside (E24) decrease the inhibitory effect. It seems that the effects of glycosylation might be diverse, depending on the structure of flavonoid aglycone, type of glycoside, as well as the position of substitution. Similar results were also reported by Gao et al., who found that apigenin, luteolin, and myricetin showed better inhibitory effect than their corresponding glycosides, whereas the inhibitory activities of isorhamnetin-3-O-rutinoside and isorhamnetin-3-O-glucoside were much lower than isorhamnetin (34). With the aid of 3D contour maps of quantitative structure–activity relationship (QSAR), they speculated that the presence of bulky groups at position 3 of ring C was beneficial to DPP-4 inhibition, while position 4' of ring B and most part of ring A favored minor groups. This may partly explain the diverse effect of glycosylation at different position on DPP-4 inhibition of flavonoid.

#### Hydrogenation of the 2,3-Double Bond in Ring C

The 2,3-double bond in conjugation with a 4-carbonyl group in ring C plays an essential role in maintaining the spatial and practically planar molecular structure of flavonoids, which had been suggested to be favorable for inhibition of several enzymes (36–38). In the present research, the diosmetin (A4), apigenin (A1), diosmin (A5), myricetin (E19), quercetin (E23), morin (E18), showed higher inhibitory activity than hesperetin (C3), naringenin (C6), hesperidin (C4), dihydromyricetin (C2), taxifolin (C8), dihydromorin (C1), respectively. This indicates that the hydrogenation of the 2,3-double bond in ring C of flavonoids was also not beneficial to DPP-4 inhibition. In a previous research by Fan et al. (35), it was also found that the inhibitory activity of apigenin was higher than that of naringenin. Similar results were also reported by Proença et al. (29), who found that apigenin and quercetin showed a inhibitory percentage of 44 ± 2% and 46 ± 2% respectively at the test concentration of 200 μM, while naringenin and taxifolin presented no inhibitory activity against DPP-4 (lower than 20% inhibition).

### Inhibition Kinetics

**Figure 1:**
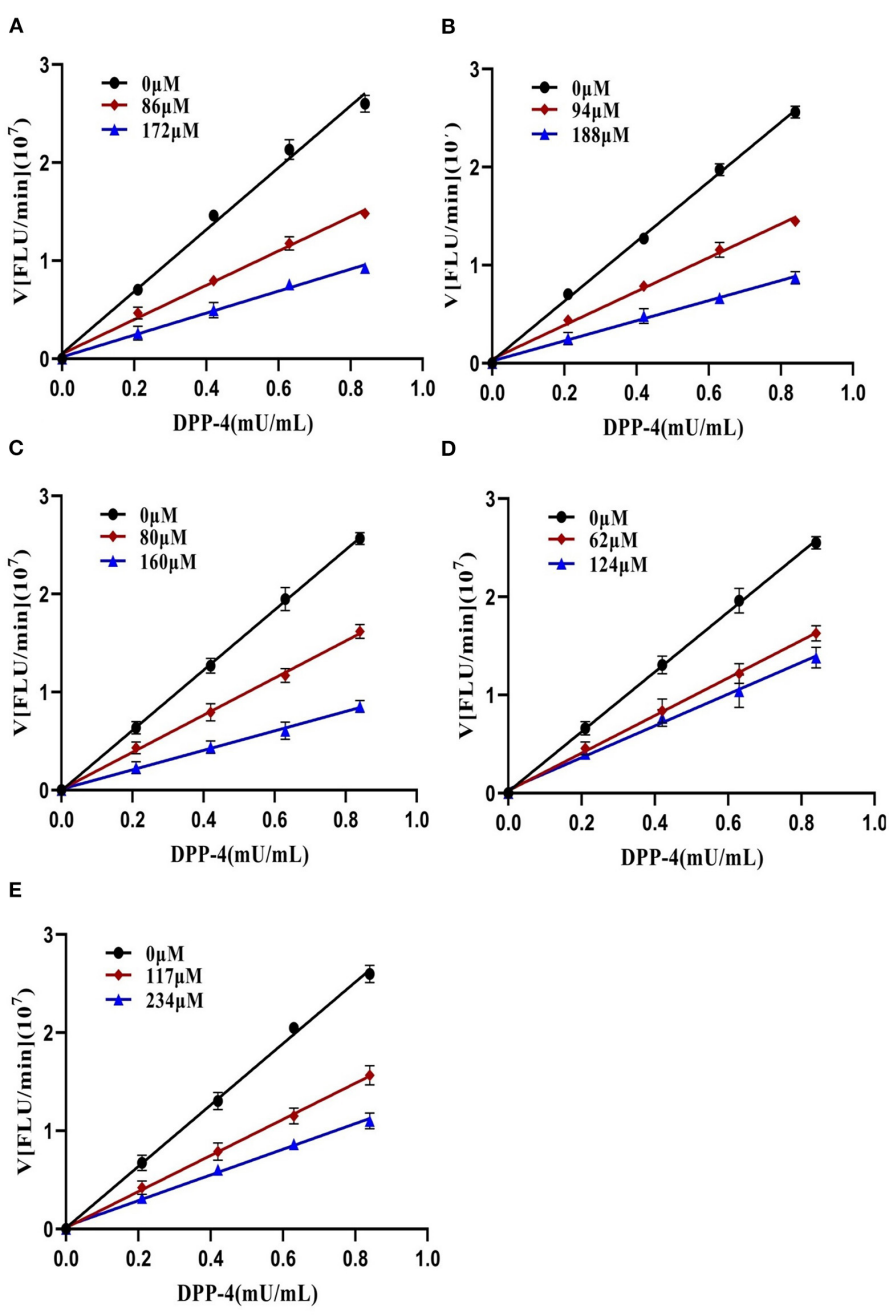
Plots of *V* vs. [DPP-4] in the absence and presence of hyperoside **(A)**, myricetin **(B)**, narcissoside **(C)**, cyanidin 3-O-glucoside **(D)**, isoliquiritigenin **(E)**.

**Figure 2:**
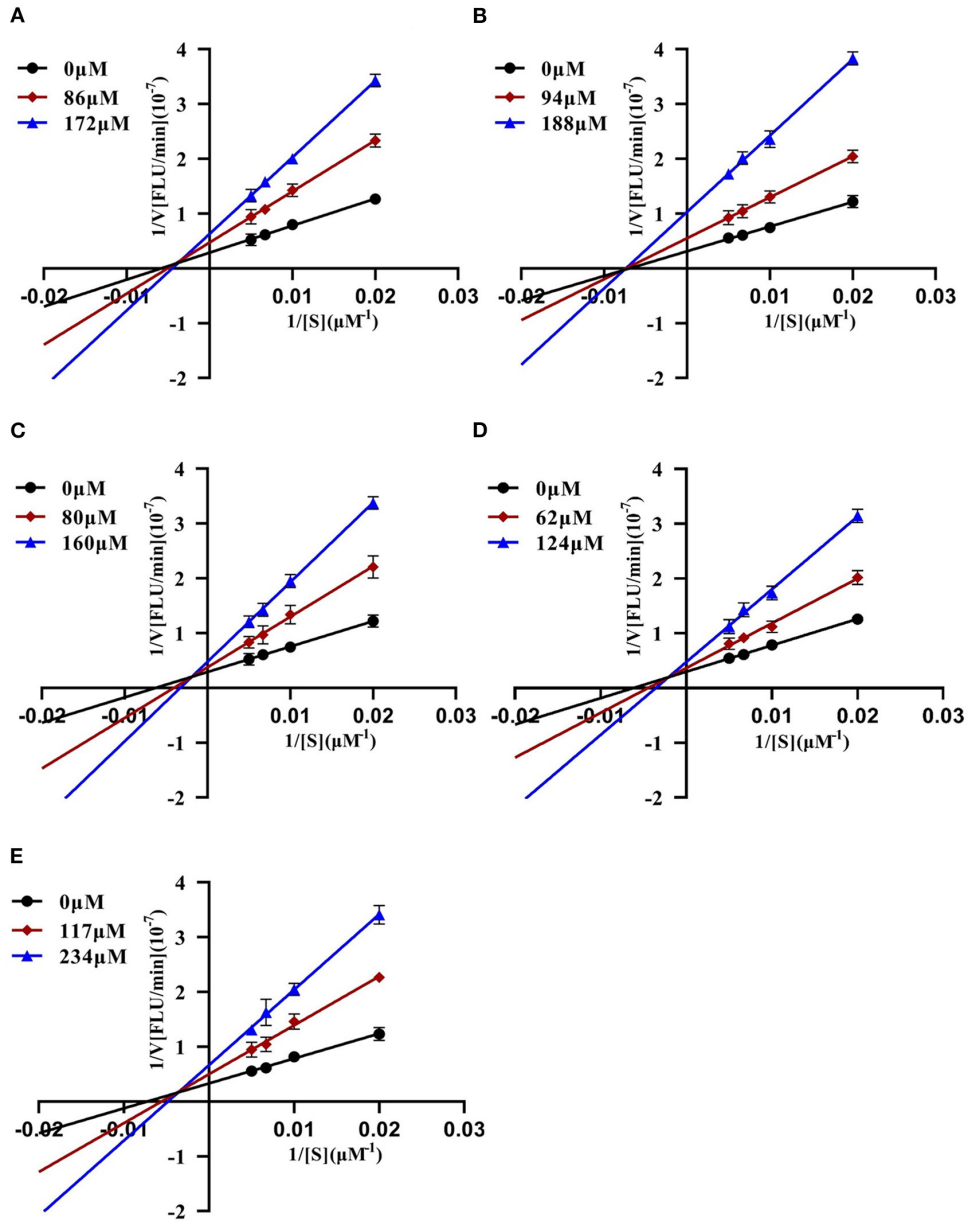
Lineweaver-Burk plots of DPP-4 inhibition by hyperoside **(A)**, myricetin **(B)**, narcissoside **(C)**, cyanidin 3-O-glucoside **(D)**, isoliquiritigenin **(E)**.

**Figure 3:**
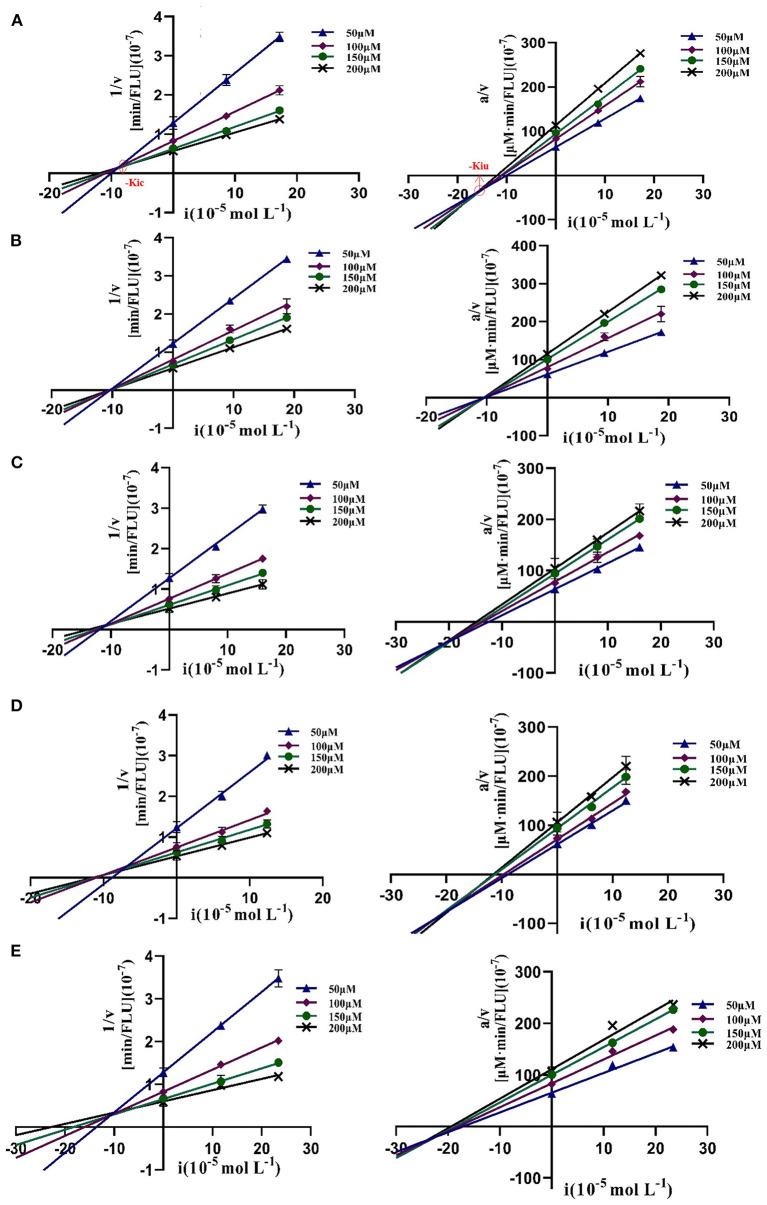
Dixon and Cornish-Bowden plots of DPP-4 inhibition by hyperoside **(A)**, myricetin **(B)**, narcissoside **(C)**, cyanidin 3-O-glucoside **(D)**, isoliquiritigenin **(E)**.

To further characterize the inhibition behavior of flavonoids against DPP-4, five flavonoids with higher inhibitory activity (IC_50_ <200 μM), including hyperoside (E4), myricetin (E19), narcissoside (E21), cyanidin 3-O-glucoside (F2), isoliquiritigenin (G1), were selected to perform the inhibition kinetic assays. The enzymatic reaction rate (*V*) vs. concentration of DPP-4 at different concentrations of inhibitor was plotted to demonstrate the reversibility of the inhibition of the above five flavonoids against DPP-4. As shown in Figure 1, the straight lines of these flavonoids at different concentrations all passed through the origins, indicating that their inhibitions on DPP-4 were all reversible. The Lineweaver-Burk plots as illustrated in Figure 2 reveal that hyperoside, narcissoside, cyanidin 3-O-glucoside, and isoliquiritigenin all inhibited DPP-4 in a mixed type due to the fact that the maximum reaction rate (*V*_*max*_) decreased and the value of Michaelis constant (*K*_*m*_) increased with the increase of inhibitor concentrations. As for myricetin, the *V*_*max*_ decreased sequentially when its concentration was increased, while the value of *K*_*m*_ remained unchanged. This indicates that the inhibition of myricetin against DPP-4 was non-competitive, which means myricetin and the substrate could bind simultaneously with the enzyme. The Dixon and Cornish-Bowden plots as illustrated in Figure 3 also demonstrated that myricetin was a non-competitive inhibitor of DPP-4 due to the fact that the intersections in both plots occurred on the *i* axis where the competitive inhibition constant (*K*_*ic*_) was equal to the uncompetitive inhibition constant (*K*_*iu*_). As for hyperoside, narcissoside, cyanidin 3-O-glucoside, and isoliquiritigenin, both the Dixon and Cornish-Bowden plots intersected at one point, which also suggests that the inhibition of DPP-4 by these flavonoids were mixed type. Furthermore, the competitive inhibition constant (*K*_*ic*_) was lower than the uncompetitive inhibition constant (*K*_*iu*_), indicating that these flavonoids preferred to insert into the active site of DPP-4 and thus exhibit more competitive inhibition patterns. Interestingly, the *K*_*ic*_ values followed the order of cyanidin 3-O-glucoside < hyperoside < isoliquiritigenin < narcissoside (Table 2), which was consistent with the order of IC_50_ values. This also indicates that the inhibition of DPP-4 by these flavonoids probably depended more on competitive type. Similar results were also reported by Fu et al., who found that the reciprocal of competitive inhibition constant (*1/K*_*ic*_) followed the same order as the inhibitory activity for the same flavonoid sort (39).

**Table 2:** Characteristic values of Dixon and Cornish-Bowden plots for the DPP-4 inhibition by hyperoside, myricetin, narcissoside, cyanidin 3-O-glucoside, and isoliquiritigenin.

**Flavonoid**	**Inhibition type**	***K_***ic***_* (10^**−5**^ mol L^**−1**^)**	***K_***iu***_*(10^**−5**^ mol L^**−1**^)**	**1/*K_***ic***_* (10^**4**^ L mol^**−1**^)**	**1/*K_***iu***_* (10^**4**^ L mol^**−1**^)**
Hyperoside	Mixed	9.03 ± 0.10	14.8 ± 0.22	1.10 ± 0.01	0.67 ± 0.01
Myricetin	Non-competitive	10.35 ± 0.11	10.35 ± 0.11	0.97 ± 0.00	1.01 ± 0.01
Narcissoside	Mixed	11.57 ± 0.21	22.3 ± 0.15	0.86 ± 0.02	0.44 ± 0.03
Cyanidin 3-O-glucoside	Mixed	7.43 ± 0.12	18.37 ± 0.24	1.30 ± 0.02	0.57 ± 0.03
Isoliquiritigenin	Mixed	9.82 ± 0.34	26.7 ± 0.34	1.01 ± 0.03	0.37 ± 0.01

### Fluorescence Quenching Analysis

#### Fluorescence Quenching Mechanism

**Figure 4:**
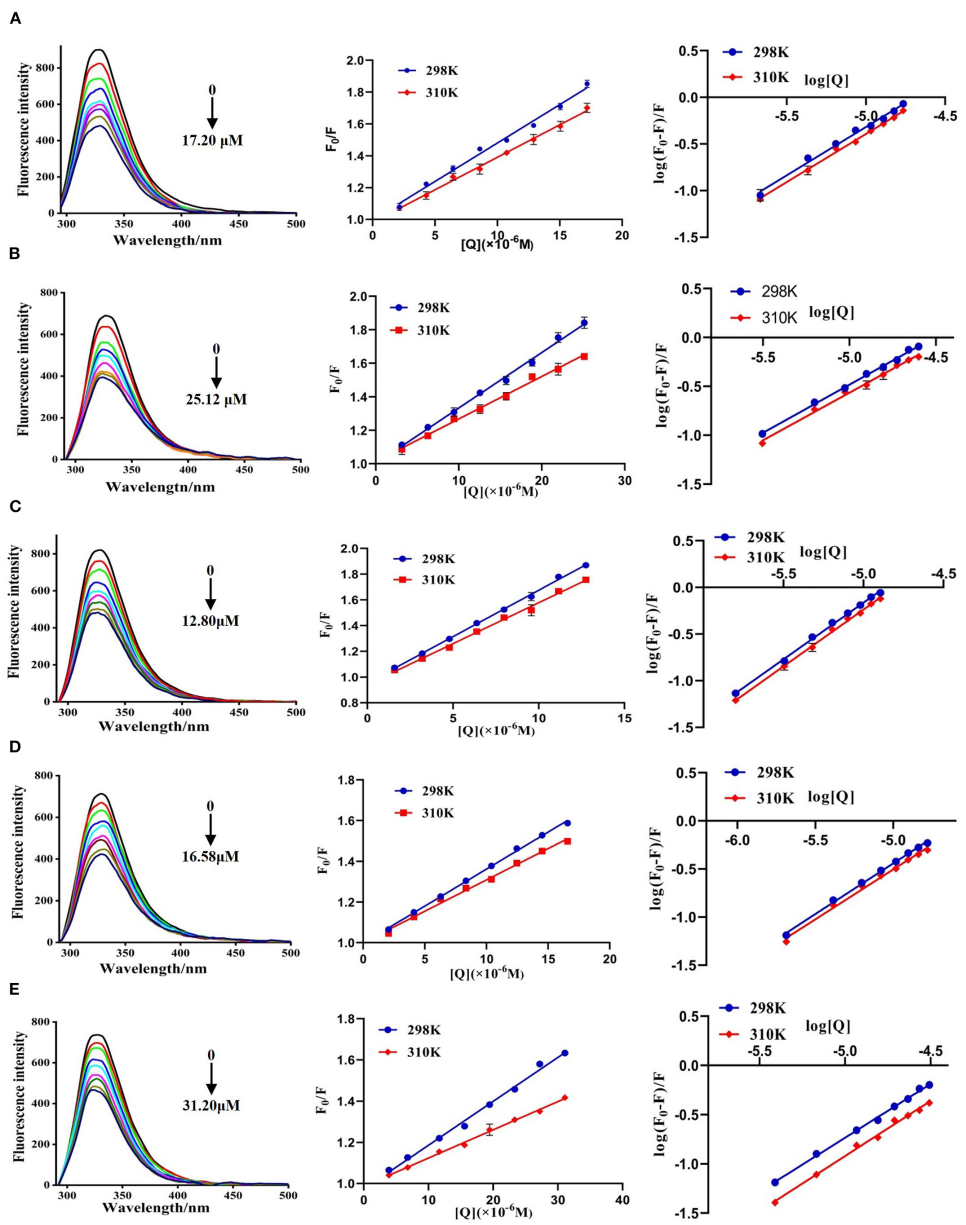
Fluorescence spectra of DPP-4 in the absence and presence of hyperoside **(A)**, myricetin **(B)**, narcissoside **(C)**, cyanidin 3-O-glucoside **(D)**, isoliquiritigenin **(E)** at different concentrations and the Stern-Volmer plots for the fluorescence quenching of DPP-4 by the flavonoids at different temperatures.

The fluorescence spectra of DPP-4 in the absence and presence of different concentrations of hyperoside, myricetin, narcissoside, cyanidin 3-O-glucoside, and isoliquiritigenin are shown in Figure 4. DPP-4 exhibited a strong emission peak around 330 nm when the excitation wavelength was set to 280 nm. With the increase of the concentration of the flavonoids, the fluorescence intensity of DPP-4 gradually decreased, indicating that binding interactions occurred between these five flavonoids and DPP-4, which led to the quenching of the intrinsic fluorescence of the enzyme.

To investigate the fluorescence quenching mechanism of these flavonoids, the fluorescence quenching data at different temperatures (298 and 310 K) was analyzed using the Stern-Volmer equation (40, 41):


$$\begin{array}{l} \frac{F_{0}}{F}\;=1+K_{SV}\left[Q\right]=1+K_{q}\tau_{0}\left[Q\right] \end{array}$$

Where *F*_0_ and *F* are the fluorescence intensities of DPP-4 in the absence and presence of flavonoid compounds, respectively. [*Q*] represents the concentration of the tested flavonoid compound. *K*_*SV*_ is the Stern-Volmer quenching constant, which could be obtained by linear regression of the plot of *F*_0_/*F* against [*Q*]. *K*_*q*_ and τ_0_ are the biomolecule quenching rate constant and average lifetime of biomolecule in the absence of the quencher (10^−8^ s), respectively. As shown in Figure 4, the plots of *F*_0_/*F* vs. [*Q*] at different temperatures had good linearity, indicating that the quenching process was a single static or dynamic quenching. Furthermore, the values of *K*_*SV*_ decreased with the increase of temperature, and the values of *K*_*q*_ were much higher than 2 × 10^10^ L mol^−1^ s^−1^ (Table 3), which is the maximum collision quenching rate constant of biological macromolecules, suggesting that the fluorescence quenching of these five flavonoids on DPP-4 was a static quenching process due to the formation of ground state complexes.

**Table 3:** Quenching constants (*K*_*sv*_), quenching rate constant (*K*_*q*_), binding constants (*K*_*b*_), number of binding sites (*n*) and thermodynamic parameters for interactions between DPP-4 and myricetin, hyperoside, narcissoside, cyanidin 3-O-glucoside, isoliquiritigenin at different temperatures.

**Flavonoid**	***T* (K)**	***K_***SV***_* (×10^**4**^ L mol^**−1**^)**	***K_***q***_* (×10^**12**^ L mol^**−1**^ s^**−1**^)**	***K_***b***_* (×10^**4**^ L mol^**−1**^)**	***n***	**Δ*G* (kJ mol^**−1**^)**	**Δ*H* (kJ mol^**−1**^)**	**Δ*S* (J mol^**−1**^K^**−1**^)**
Hyperoside	298	4.83 ± 0.01	4.83 ± 0.01	7.32 ± 0.014	1.04 ± 0.02	−27.754	−14.148	45.66
	310	4.08 ± 0.01	4.08 ± 0.01	5.87 ± 0.013	1.03 ± 0.02	−28.301		
Myricetin	298	3.305 ± 0.06	3.305 ± 0.06	2.76 ± 0.011	0.98 ± 0.02	−25.34	−36.52	−37.53
	310	2.517 ± 0.05	2.517 ± 0.05	1.56 ± 0.012	0.95 ± 0.02	−24.88		
Narcissoside	298	7.212 ± 0.01	7.212 ± 0.01	81.28 ± 0.013	1.22 ± 0.02	−33.72	−8.105	85.93
	310	6.389 ± 0.01	6.389 ± 0.01	71.61 ± 0.012	1.21 ± 0.02	−34.75		
Cyanidin 3-O-glucoside	298	3.64 ± 0.04	3.64 ± 0.04	6.67 ± 0.01	1.05 ± 0.01	−27.52	−8.246	64.68
	310	3.08 ± 0.06	3.08 ± 0.06	5.86 ± 0.013	1.04 ± 0.01	−27.79		
Isoliquiritigenin	298	2.133 ± 0.04	2.133 ± 0.04	5.38 ± 0.011	1.09 ± 0.01	−26.99	−7.952	63.88
	310	1.37 ± 0.03	1.37 ± 0.03	4.75 ± 0.012	1.12 ± 0.02	−27.76		

#### Binding Constant and Number of Binding Sites

For the static fluorescence quenching process, the binding constant (*K*_*b*_) and number of binding sites (*n*) between the flavonoids and DPP-4 can be calculated according to the following equation (40, 41):


$$\begin{array}{c} log\frac{F_{0}-F}{F}=logK_{b}+nlog\left[\text{Q}\right] \end{array}$$

Where *F*_0_ and *F* are the fluorescence intensities of DPP-4 in the absence and presence of flavonoid compounds, respectively. [*Q*] represents the concentration of flavonoid compound. *K*_*b*_ and *n* are the binding constant and number of binding sites, respectively.

The plots of log[(*F*_0_-*F*)/*F*] vs. log[*Q*] for the fluorescence quenching of myricetin, hyperoside, narcissoside, cyanidin 3-O-glucoside, and isoliquiritigenin on DPP-4 are shown in Figure 4, and the values of *K*_*b*_ and *n* are listed in Table 3. All the tested flavonoid compouds showed a *K*_*b*_ value of higher than 10^4^ L mol^−1^, indicating that there existed strong binding interactions between these flavonoid compounds and DPP-4. Among them, narcissoside showed the highest *K*_*b*_ value, suggesting that it had the strongest affinity ability for DPP-4. The binding constants (*K*_*b*_) of the five flavonoid compounds were all decreased with elevation of temperature, indicating the formation of unstable complexes between these flavonoids and DPP-4. These results also proved that the fluorescence quenching of these flavonoids on DPP-4 was a static process. The number of binding sites (*n*) between these flavonoids and DPP-4 were approximately equal to 1, suggesting that there was only one binding site on DPP-4 for these flavonoid compounds.

#### Thermodynamic Parameters

To ascertain the intermolecular interaction forces between the flavonoids and DPP-4, the thermodynamic parameters (enthalpy change, Δ*H*; entropy change, Δ*S*; Gibbs free-energy change, Δ*G*) were calculated according to the following equations (40, 42):


$$\begin{array}{c} ln\left(K_{2}/K_{1}\right)=\left(1/T_{1}-1/T_{2}\right)\times \Delta H/R\\ \Delta G=-RTlnK_{b}\\ \Delta S=\left(\Delta H-\Delta G\right)/T \end{array}$$

Where *R* is the gas constant (8.314 J mol^−1^ K^−1^), and *K*_*b*_ is the binding constant at the corresponding temperature (*T*).

As shown in Table 3, all the values of Gibbs free-energy change (Δ*G*) were negative, indicating that the five flavonoids could bind with DPP-4 spontaneously. Both the values of enthalpy change (Δ*H*) and entropy change (Δ*S*) of the binding reaction between myricetin and DPP-4 were negative, which suggests that hydrogen bonds and van der Waals were the predominant forces to maintain the complex of myricetin with DPP-4 according to a previous report by Ross & Subramanian (43). The interactions between hyperoside, narcissoside, cyanidin 3-O-glucoside, isoliquiritigenin and DPP-4 showed a negative Δ*H* value and a positive Δ*S* value, indicating that electrostatic forces might play an important role in stabilizing the complexes of these flavonoids with DPP-4. Nevertheless, the negative Δ*H* value cannot be mainly ascribed to electrostatic interactions due to the fact that the Δ*H* value for these interactions is very small, almost zero, and a negative Δ*H* value can be observed whenever hydrogen bonding occurs in the binding interaction (43). Furthermore, the positive Δ*S* value is usually considered as the evidence of hydrophobic interactions (44). Therefore, hydrogen bonding and hydrophobic forces might also play an important role in the binding of hyperoside, narcissoside, cyanidin 3-O-glucoside, isoliquiritigenin to DPP-4, which would be confirmed by the molecular docking results. Interestingly, the values of /Δ*G*/, *Ksv, K*_*q*_, and *K*_*b*_ of the four flavonoids that exhibited mixed inhibition type followed the same order of narcissoside>hyperoside>cyanidin 3-O-glucoside>isoliquiritigenin, indicating that higher/Δ*G*/facilitated the binding of these flavonoids to DPP-4 and thus the fluorescence quenching effects were enhanced. However, myricetin, which exhibited the non-competitive inhibition type, showed a higher value of *Ksv* than isoliquiritigenin, whereas the values of /Δ*G*/ and *K*_*b*_ for myricetin were lower than those for isoliquiritigenin. This indicates that the correlation between /Δ*G*/ and fluorescence quenching effect might be affected by the inhibition type and interaction mode. Further researches might be essential to elucidate the mechanisms involved.

#### Synchronous Fluorescence Spectra

**Figure 5:**
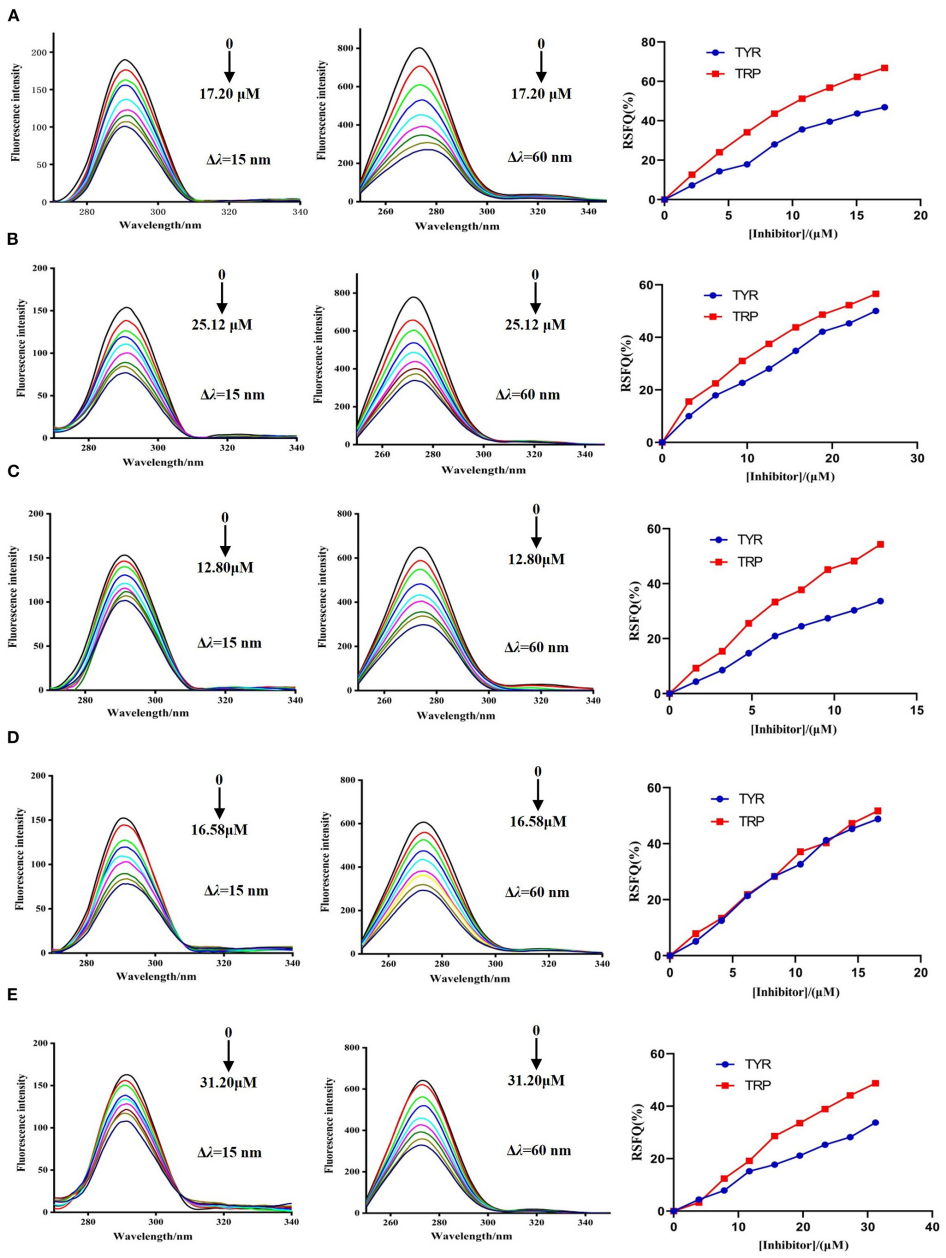
The synchronous fluorescence spectra of DPP-4 and RSFQ values in the absence and presence of hyperoside **(A)**, myricetin **(B)**, narcissoside **(C)**, cyanidin 3-O-glucoside **(D)**, isoliquiritigenin **(E)** at different concentrations.

The synchronous fluorescence spectra of DPP-4 in the presence of myricetin, hyperoside, narcissoside, cyanidin 3-O-glucoside, and isoliquiritigenin at different concentrations were measured to investigate the microenvironmental changes of the tyrosine (Tyr) and tryptophan (Trp) residues in DPP-4 upon interacting with the flavonoids. As shown in Figure 5, with the increase of the concentration of the tested flavonoids, the fluorescence intensities of tyrosine residue (Δλ = 15 nm) and tryptophan (Δλ = 60 nm) in DPP-4 gradually weakened, and the ratios of synchronous fluorescence quenching (RSFQ) of tryptophan residues was higher than those of tyrosine residues, which suggests that the tryptophan residues are the main fluorescence quenching group for the flavonoids and the binding site of them in DPP-4 might be closer to tryptophan residues. No obvious shift could be observed for the fluorescence peak of tyrosine residue after interacting with the flavonoids, indicating that all the tested flavonoids had little effect on the microenvironment around tyrosine residues in DPP-4. However, the fluorescence peak of tyrosine residues showed a slight red shift when interacting with the tested flavonoids, indicating that the hydrophobicity of the environment around tryptophan residues in DPP-4 was decreased and thus the conformation of DPP-4 might change.

### Molecular Docking

**Figure 6:**
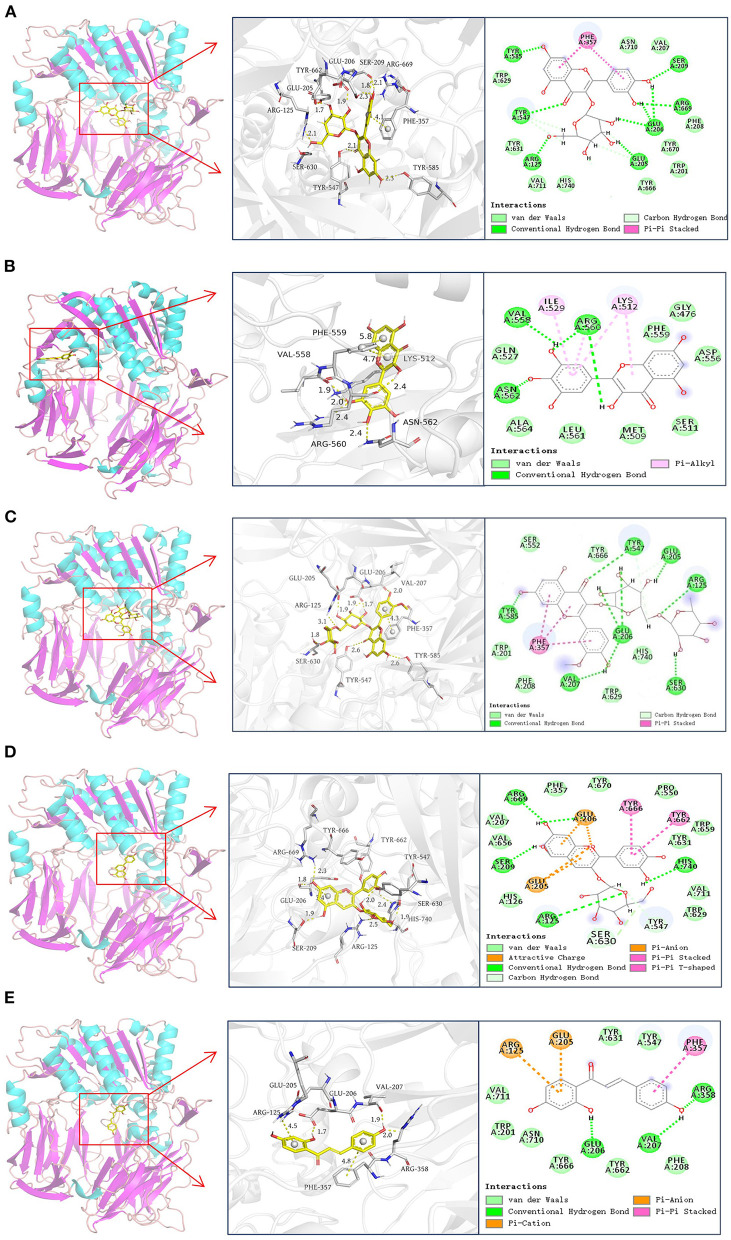
Docking results of hyperoside **(A)**, myricetin **(B)**, narcissoside **(C)**, cyanidin 3-O-glucoside **(D)**, isoliquiritigenin **(E)** with DPP-4.

Molecular docking was used to simulate the binding interactions between DPP-4 and hyperoside, myricetin, narcissoside, cyanidin-3 O-glucoside, isoliquiritigenin, and the best docking results are illustrated in Figure 6 and Table 4.

**Table 4:** Docking results of hyperoside, myricetin, narcissoside, cyanidin 3-O-glucoside, and isoliquiritigenin with DPP-4.

**Flavonoid**	**Binding energy (kcal mol^**−1**^)**	**Interacting residues**	**Hydrogen bonded residues**
Hyperoside	−8.12	Arg125, Trp201, Glu205, Glu206, Ser209, Val207, Phe208, Phe357, Tyr547, Tyr585, Trp629, Arg669, Val711	Arg125, Glu205, Glu206, Ser209, Tyr547, Tyr585, Arg669
Myricetin	−8.03	Pro475, Met509, Pro510, Ser511, Lys512, Gln527, Ile529, Val558, Phe559, Arg560, Ala564, Asn562	Val558, Arg560, Asn562
Narcissoside	−9.38	Arg125, Trp201, Glu205, Glu206, Val207, Phe208, Phe357, Tyr547, Ser552, Tyr585, Trp629, Ser630, Tyr666, His740	Arg125, Glu205, Glu206, Val207, Tyr547, Tyr585
Cyanidin-3-O-glucoside	−8.68	Arg125, His126, Glu205, Glu206, Val207, Ser209, Phe357, Pro550, Tyr547, Trp629, Ser630, Tyr631, Val656, Trp659, Tyr662, Tyr666, Arg669, Tyr670, Val711, His740	Arg125, Glu206, Ser209, Tyr547, Ser630, Arg669, His740
Isoliquiritigenin	−7.05	Arg125, Trp201, Glu205, Glu206, Val207, Phe208, Phe357, Tyr547, Tyr631, Tyr662, Tyr666, Asn710, Val711	Glu206, Val207, Arg358

According to previous reports (45–48), the active site of DPP-4 consists of a catalytic triad (Ser630, Asp708, and His740), an oxyanion hole containing Tyr547 and Ser631, a highly hydrophobic pocket S1 formed by the side chains of Tyr631, Val656, Trp659, Tyr662, Tyr666, and Val711, and a region with saline bridging amino residues such as Glu205, Glu206 and Tyr662. Several other amino acid residues, including Arg125, Val207, Ser209, Phe357, Arg358, Tyr547, Gly628, Gly632, and Asn710, are also involved in the ligand binding and catalytic mechanism of DPP-4 (45, 46, 48–50). As shown in Figure 6 and Table 4, hyperoside, narcissoside, cyaniding 3-O-glucoside, and isoliquiritigenin inserted into the active site cavity of DPP-4 and interacted with the key amino acid residues of the active site, whereas the binding site of myricetin was located in a minor cavity close to the active pockets of DPP4, exhibiting a non-competitive binding mode.

Hyperoside bound to the active pocket of DPP-4 with a binding energy of −8.12 kcal/mol, which was also lower than that obtained from fluorescence quenching analysis. Nine hydrogen bonds were generated between hyperoside and the amino acid residues Glu205, Glu206, Arg669, Ser209, Tyr585, Tyr547, and Arg125 with a distance of 1.7Å, 1.9Å, 1.8Å, 2.1Å, 2.3Å, 2.5Å, 2.3Å, 2.1Å, and 2.1Å, respectively, which played an important role in stabilizing the complex of hyperoside with DPP-4. Additionally, hyperoside also interacted with the hydrophobic amino acid residues of Phe357, Val207, Phe208, Trp201, Val711, Trp629, and a π-π stacked interaction was found between Phe357 and the aromatic ring A and B of hyperoside, which might enhance the stability of the complex.

Myricetin was surrounded by Val558, Ile529, Lys512, Phe559, Pro475, Ser511, Met509, Pro510, Arg560, Ala564, Asn562, Gln527. Among them, Val558 and Asn562 interacted with myricetin by forming hydrogen bonds with the hydroxyl groups at C3' and C4' with a distance of 1.9 Å and 2.4 Å, respectively. Arg560 interacted *via* two hydrogen bonds with two hydroxyl groups at C3'and C3 of mycretin with a distance of 2.0 Å and 2.4Å, respectively. Ile529, Lys512, and Arg560 exhibited π-alkyl hydrophobic interactions with myricetin. Other amino acid residues mainly interacted with myricetin through van der Waals forces. The binding energy was −8.03 kcal mol^−1^, which was lower than that obtained from fluorescence quenching analysis. Other research also observed this difference, which could be probably owing to the presence of dissolving solvent energy in the reaction process of fluorescence quenching (33).

Similar to hyperoside, narcissoside also bound to the active site of DPP-4 with a lower binding energy (−9.38 kcal/mol) than that obtained from fluorescence quenching analysis. Its hydroxyl groups at C7, C4' and carbonyl group at C4 interacted with Tyr585, Glu206, Val207, and Tyr547 through hydrogen bonds at a distance of 2.6Å, 2.0Å, 2.0Å, and 2.6Å, respectively. Moreover, the hydroxyl groups on the rutinoside substituent at C3 position also formed five hydrogen bonds with Arg125, Glu205, Glu206, and Ser630 at a distance of 3.1Å, 1.9Å, 1.9Å, 1.7Å, and 1.8Å, respectively. These findings confirmed the speculation that the increasing number of hydroxyl groups generated by glycoside substituent could enhance the binding affinity of flavonoids to the enzyme and partly explained the higher inhibitory activity of narcissoside than isorhamnetin. The hydrophobic interactions with Trp201, Trp629, Val207, Phe208, Phe357, and the π-π stacked interactions with the benzene ring of Phe357 as well as the van der Waals interactions with Trp201, Phe208, Ser552, Trp629, Tyr666, His740, also contributed to the conformational stability of the complex.

The binding site of cyanidin 3-O-glucoside mainly consisted of Arg125, His126, Glu205, Glu206, Val207, Ser209, Phe357, Pro550, Tyr547, Trp629, Ser630, Tyr631, Val656, Trp659, Tyr662, Tyr666, Arg669, Tyr670, Val711, and His740. The free hydroxyl groups on the ring A and C4' position of ring B of cyaniding 3-O-glucoside generated four hydrogen bonds with Ser209, Arg669, Glu206, and His740 at a distance of 1.9Å, 2.3Å,1.8Å, and 1.9Å, respectively. Similar to narcissoside, the hydroxyl group on the glucoside substituent at C3 position also formed a hydrogen bond with Arg125 at a distance of 2.5Å. Furthermore, two carbon hydrogen bonds with Tyr547 and Ser630, two π-π stacked interactions with Tyr666 and Tyr662, two π-anion interactions with Glu205 and Glu206, and two attractive charge interactions with Glu205 and Glu206, were observed from the docking results, which might play important roles in maintaining the binding conformation of the cyanidin 3-O-glucoside-DPP-4 complex. The remaining amino acid residues mainly interacted with cyanidin 3-O-glucoside through van der Waals forces.

The binding energy of isoliquiritigenin was −7.05 kcal/mol, which was also lower than that obtained from fluorescence quenching analysis. It interacted with the key amino acid residues in the active pocket of DPP-4, including Glu206, Val207, and Arg358, by forming hydrogen bonds with its hydroxyl groups at C4' and C2 with a distance of 1.7Å, 1.9Å, and 2.0Å, respectively. A π-π stacked interaction between the aromatic ring B of isoliquiritigenin and the key amino acid residue Phe357 of DPP-4, a π-anion interaction with Glu205, and a π-cation interaction with Arg125, were also observed. Several other amino acid residues, such as Trp201, Phe208, Tyr547, Tyr631, Tyr662, Tyr666, Asn710, and Val711, showed van der Waals interactions with isoliquiritigenin. These interactions together enhance the binding stability of isoliquiritigenin to DPP-4.

## Conclusion

A pannel of 70 structurally diverse flavonoids was used to evaluate their inhibitory activities against DPP-4, among which myricetin, hyperoside, narcissoside, cyanidin 3-O-glucoside, and isoliquiritigenin, showed higher inhibitory activities in a concentration-dependent manner. Structure-activity relationship analysis revealed that introducing hydroxyl groups to the positions 3' and 4' of ring B as well as the position 6 of ring A in the flavonoid structure was beneficial to improving the inhibitory efficacy against DPP-4, whereas the hydroxylation at position 3 of ring C in the flavonoid structure was unfavorable for the inhibition. Besides, the methylation of the hydroxyl groups at C3', C4', and C7 of the flavonoid structure tended to lower the inhibitory activity against DPP-4, and the 2,3-double bond and 4-carbonyl group on ring C of the flavonoid structure was essential for the inhibition. Glycosylation affected the inhibitory activity diversely, depending on the structure of flavonoid aglycone, type of glycoside, as well as the position of substitution. Among the five flavonoids with higher inhibitory activity against DPP-4, myricetin showed a non-competitive inhibition mode toward DPP-4, whereas hyperoside, narcissoside, cyanidin 3-O-glucoside, and isoliquiritigenin all reversibly inhibited DPP-4 in a mixed type. Moreover, the fluorescence quenching analysis indicated that all the five flavonoid compounds could effectively quench the intrinsic fluorescence of DPP-4 by spontaneously binding with DPP-4 to form unstable complexes. Hydrogen bonds and van der Waals were the predominant forces to maintain the complex of myricetin with DPP-4, and electrostatic forces might play an important role in stabilizing the complexes of the remaining four flavonoids with DPP-4. The binding of the tested flavonoids to DPP-4 could also induce the conformation change of DPP-4 and thus led to inhibition on the enzyme. Molecular docking simulation further ascertained the binding interactions between DPP-4 and the selected five flavonoids, among which hyperoside, narcissoside, cyanidin 3-O-glucoside, and isoliquiritigenin inserted into the active site cavity of DPP-4 and interacted with the key amino acid residues of the active site, whereas the binding site of myricetin was located in a minor cavity close to the active pockets of DPP-4. This research gave an insight into the structural requirements of flavonoids to inhibit DPP-4 by using a panel of 70 structurally diverse flavonoids and elucidated the interaction mechanisms between the selected flavonoids and DPP-4, which not only is beneficial to screening and structural modification of flavonoids as potential DPP-4 inhibitors, but also can promote the application of natural dietary flavonoids for development of nutraceutical products.

## Data Availability Statement

The original contributions presented in the study are included in the article/supplementary material, further inquiries can be directed to the corresponding author.

## Author Contributions

JP, QZ, and ZJ designed the experiments and analyzed the data. JP performed the experiments with the aid from QZ, CZ, WY, ZL, HL, and JL. JP drafted the manuscript with the aid from QZ and ZJ. ZJ reviewed and supervised the final version of the manuscript. All authors reviewed the manuscript and contributed to the article and approved the submitted version.

## Funding

This work was funded by the Agricultural Science and Technology Innovation Program (ASTIP) of the Chinese Academy of Agricultural Sciences (Grant No. CAAS-ASTIP-ZFRI) and the Key Science and Technology Research Project of Henan Province (Grant No. 202102110209).

## Conflict of Interest

The authors declare that the research was conducted in the absence of any commercial or financial relationships that could be construed as a potential conflict of interest.

## Publisher's Note

All claims expressed in this article are solely those of the authors and do not necessarily represent those of their affiliated organizations, or those of the publisher, the editors and the reviewers. Any product that may be evaluated in this article, or claim that may be made by its manufacturer, is not guaranteed or endorsed by the publisher.
